# Plasma Lipidome, *PNPLA3* polymorphism and hepatic steatosis in hereditary hemochromatosis

**DOI:** 10.1186/s12876-020-01282-3

**Published:** 2020-07-17

**Authors:** Jessica Seeßle, Hongying Gan-Schreier, Marietta Kirchner, Wolfgang Stremmel, Walee Chamulitrat, Uta Merle

**Affiliations:** 1 Department of Gastroenterology, University Hospital Heidelberg, Im Neuenheimer Feld 410, 69120 Heidelberg, Germany; 2 Institute of Medical Biometry and Informatics, University Hospital Heidelberg, Heidelberg, Germany

**Keywords:** Hereditary hemochromatosis, Plasma lipidome, Phospholipids, Triglycerides, Nonalcoholic fatty liver disease, *PNPLA3* polymorphism

## Abstract

**Background:**

Hereditary hemochromatosis (HH) is an autosomal recessive genetic disorder with increased intestinal iron absorption and therefore iron Overloa*d. iron* overload leads to increased levels of toxic non-transferrin bound iron which results in oxidative stress and lipid peroxidation. The impact of iron on lipid metabolism is so far not fully understood. The aim of this study was to investigate lipid metabolism including lipoproteins (HDL, LDL), neutral (triglycerides, cholesterol) and polar lipids (sphingo- and phospholipids), and *PNPLA3* polymorphism (rs738409/I148M) in HH.

**Methods:**

We conducted a cohort study of 54 subjects with HH and 20 healthy subjects. Patients were analyzed for their iron status including iron, ferritin, transferrin and transferrin saturation and serum lipid profile on a routine follow-up examination.

**Results:**

HH group showed significantly lower serum phosphatidylcholine (PC) and significantly higher phosphatidylethanolamine (PE) compared to healthy control group. The ratio of PC/PE was clearly lower in HH group indicating a shift from PC to PE. Triglycerides were significantly higher in HH group. No differences were seen for HDL, LDL and cholesterol. Hepatic steatosis was significantly more frequent in HH. *PNPLA3* polymorphism (CC vs. CG/GG) did not reveal any significant correlation with iron and lipid parameters including neutral and polar lipids, grade of steatosis and fibrosis.

**Conclusion:**

Our study strengthens the hypothesis of altered lipid metabolism in HH and susceptibility to nonalcoholic fatty liver disease. Disturbed phospholipid metabolism may represent an important factor in pathogenesis of hepatic steatosis in HH.

## Background

Hereditary hemochromatosis (HH) is an autosomal recessive genetic disorder with increased intestinal iron absorption which leads to iron overload and consequently to tissue damage and functional impairment of organs like liver, pancreas, and heart [1–3]. Iron is an essential metal involved in a wide spectrum of physiological functions such as oxygen transport and enzymatic reactions. Iron overload leads to increased levels of toxic non-transferrin bound iron (NTBI) which results in oxidative stress and lipid peroxidation [3, 4].

Iron overload has a direct effect on hepatic lipid metabolism [5]. Animal models with dietary iron overload showed marked changes in plasma lipid profiles with elevated triglycerides and total cholesterol, decreased high-density lipoprotein (HDL), altered composition of very-low density lipoprotein (VLDL) and hepatic sterol metabolism [6–8]. Iron treated HepG2 cells revealed a decrease of apolipoprotein B100 (ApoB100) and VLDL secretion [9] which was reversed by the iron chelator desferroxamine [10]. Additionally, iron overload increased intracellular lipid droplets, which was associated with increased phosphatidylserine (PS) in the outer leaflet of the plasma membrane [11]. This alteration was also observed in membranes of erythrocytes in HH [12]. Iron excess induces distinct changes in the serum concentrations of unsaturated long-chain fatty-acyl phosphatidylcholine (PC) including PC 40:2, PC 40:3, PC 40:4, and PC 42:1 in diabetes mellitus type 2 [13]. Inhibition of hepatic PC biosynthesis results in triglyceride accumulation and impairs the secretion of VLDL [14, 15]. In rat livers excess dietary iron intake causes pro-steatotic state which was associated with a decrease in ω-3 long-chain polyunsaturated fatty acid (PUFA) levels and an upregulation of the expression of lipogenic transcription factors [16].

In humans iron overload causes elevated triglycerides [17]. Genetic predisposition for HH is associated with primary hypertriglyceridemia [18]. Hypertriglyceridemia was found in almost a third of subjects with HH which was significantly decreased by phlebotomies [19] whereas in non-HH subjects with hyperferritinemia and hypertriglyceridemia repeated phlebotomies did not reduce triglyceride concentrations [20]. Reduced levels of LDL at baseline [10] and an increase of HDL under phlebotomy in HH patients were shown [21], but in their study no effect of phlebotomy was seen on total cholesterol, low density lipoprotein (LDL) and triglyceride levels [21]. Hence, iron has effects on hepatocellular metabolism of phospholipids and lipoproteins, and on unsaturation of fatty acids in experimental and clinical settings.

Obesity-related steatosis is already identified as cofactor in liver injury in HH [22], but HH is also associated with increased susceptibility to nonalcoholic fatty liver disease (NAFLD) despite lower BMI and lower triglycerides levels [23, 24]. Nevertheless the role of HFE polymorphism in the occurrence of NAFLD is not fully understood.

*PNPLA3* (human patatin like phospholipase domain-containing 3) belongs to a group of lipid metabolizing enzymes [25]. In humans, *PNPLA3* has the highest expression in the hepatic stellate cells, retina, and hepatocytes [26]. The rs738409 CG variant in *PNPLA3* is considered the major genetic determinant of NAFLD [27]. SNPs rs738409 C > G in the *PNPLA3* gene encodes for the isoleucine to methionine substitution at position 148 (I148M). It has been shown that *PNPLA3* has a triglyceride hydrolase activity [28]. The I148M mutation causes a loss of function of the enzymes activity leading to impairment of lipid catabolism, lipid droplets remodeling, and VLDL secretions in hepatocytes with reduced fatty acid hydrolysis, increased triglyceride accumulation and a decrease of VLDL secretion thereby contributing to hepatic steatosis, inflammation and a greater risk for developing fibrosis [26, 29–32]. These alterations were observed with and without symptoms of metabolic syndrome like insulin resistance and obesity [33, 34]. In HH the I148M variant was linked with the severity of fibrosis [35]. The mechanism underlying the progression of liver disease is still under investigation. It is hence surmised that the role of iron overload on hepatocellular lipid metabolism may involve *PNPLA3*.

Therefore, the aim of our study was to evaluate alterations in lipid metabolism including analyses of blood neutral and polar lipids depending on iron status and *PNPLA3* polymorphism (rs738409/I148M) in our HH cohort in comparison to healthy control group.

## Methods

### Study population

A total of 54 subjects with HH were retrospectively examined at Internal Medicine IV, University Heidelberg Hospital from 2012 to 2019. Written informed consent was obtained from each patient. Ethical approval was given by the Ethics Committee of University of Heidelberg. Results of HH patients were compared with 20 healthy control subjects. The study protocol was approved by the ethical committee of the University of Heidelberg. Patients serum samples were collected on a routine follow-up examination for HbA1c, BMI, albumin, bilirubin, GGT, AST, ALT, prothrombin time (P), ferritin, transferrin, transferrin saturation (TS), iron, HFE-genotype, and I148M *PNPLA3* polymorphism (rs738409/I148M) as well as serum lipids and lipoproteins including triglycerides, cholesterol, HDL, LDL and phospho- and sphingolipids. Patients with alcohol consumption > 20 g/day and statin therapy or any other lipid lowering therapy were excluded from the study. Ultrasound and evaluation for liver fibrosis using noninvasive method (Fibroscan) was performed by experienced medical doctors. The duration of experience was more than six months as a fulltime job in the department of sonography. Sonographic features to evaluate patients for hepatic steatosis were increased echogenicity of the background liver parenchyma, difference in echogenicity between the liver and right kidney, obscuration of the margins of the portal triads within the liver, and attenuation of the sound beam with diminished visualization of the deep right lobe. These criteria were used in the assessment of the presence and graduation of hepatic steatosis as normal, mild, moderate and severe. Diagnosis of HH was done by genetic analysis (C282Y) in the hospital central laboratory according to standard methods and determination of iron parameters (iron, ferritin, transferrin, TS). Patients with HH were treated by phlebotomy. The therapy was monitored by the determination of ferritin which should be less than 100 μg/l.

### Lipidomic analysis

Serum samples were subjected to lipid extraction according to Folch methods. The levels of phospho- and sphingolipids in lipid extracts were determined with a triple-quadrupole Micro Mass Quattro Premier mass spectrometer coupled with a liquid-chromatography system using the running conditions as previously described [36]. The following polar lipids (phospho- and sphingolipids) were measured: phosphatidylcholine (PC), lysophosphatidylcholine (LPC), phosphatidylinositol (PI), phosphatidylserine, phosphatidylethanolamine (PE), lysophosphatidylethanolamine (LPE), and sphingomyelin (SM). Serum polar lipids were quantified as ng/μl serum.

### Statistical methods

Variables were described by mean ± standard deviation (SD) or median (interquartile range IQR) or frequencies, respectively. By means of detailed summary statistics and visual inspection of the distribution normality assumption was checked. Statistical differences in means between two groups (healthy vs. HH and CC vs. CG/GG) were evaluated using Welch’s t-test (due to unequal sample size homogeneity of variances cannot be assumed) or the nonparametric Mann-Whitney U test in case normal distribution cannot be assumed. Statistical differences in proportions were assessed by chi-square test or Fisher’s exact test in case of small expectancy counts. All analyses were performed with SPSS version 24 (IBM SPSS Statistic, Chicago, IL, USA). Two-sided *p*-values were interpreted descriptively and a p-value of < 0.05 was considered statistically significant.

## Results

### Baseline characteristics of study population and stratified by low and high transferrin saturation

Patient characteristics are outlined in Table 1. A total of 54 subjects with HH were analyzed and compared with 20 healthy subjects. The two groups were significantly different in serum iron contents (Healthy subjects vs. HH: transferrin 2.1 vs. 1.8 g/l, *p* < 0.0001, transferrin saturation 26.0% vs. 70.1%, *p* < 0.0001, iron 13.5 μmol/l vs. 31.8 μmol/l, *p* < 0.0001, ferritin 133.0 μ/l vs. 261.0 μ/l, *p* = 0.02). Triglycerides (74.0 vs. 103.5 mg/dl, *p* = 0.01) were significantly higher in HH group. Cholesterol, HDL and LDL did not differ between the groups. GGT (12.5 mg/dl vs. 23.0 mg/dl, *p* = 0.001) and AST (22.0 U/l vs. 29.0 U/l, *p* = 0.01) were in the normal range but significantly increased in HH group. Sonography of the liver showed that 29.6% of subjects with HH had normal liver without hepatic steatosis while 33.3, 22.2 and 9.3% had mild, moderate and severe steatosis, respectively. Significant more hepatic steatosis in HH compared to the control group was found (*p* < 0.0001). Fibroscan measured liver stiffness of 6.7 kPa (± 4.8 kPa) in HH.

**Table 1:** Clinical characteristics of study population. Mean ± SD or median (IQR) or frequencies *n* (%) are shown with *p*-values for the group differences based on Welch’s t-test (a), Mann-Whitney U test (b), chi-square test (c) or Fisher’s exact test (d)

	Limits of normal	Healthy *n* = 20	HH *n* = 54	***p***-value
**gender (male)**		11 (55%)	39 (72.2%)	0.98^c^
**Age (years)**		48.9 ± 11.8	51.3 ± 15.0	0.47^a^
**Type II diabetes mellitus**		0 (0%)	6 (11.1%)	0.18^d^
**BMI**	kg/m^2^	24.4 ± 2.9	26.0 ± 3.4	0.05^a^
**Cholesterol**	mg/dl	186.6 ± 35.5	188.0 ± 38.3	0.89^a^
**HDL**	> 40 mg/dl	60.0 ± 17.3	55.3 ± 19.2	0.34^a^
**LDL**	< 160 mg/dl	109.4 ± 31.1	111.7 ± 26.8	0.79^a^
**Triglycerides**	< 150 mg/dl	74.0 (48.0)	103.5 (83.0)	**0.01**^**b**^
**Albumin**	30–50 g/l	44.6 ± 1.5	44.8 ± 2.6	0.75^a^
**Bilirubin**	< 1.0 mg/dl	0.6 (0.2)	0.8 (0.6)	0.38^b^
**GGT**	< 60 U/l	12.5 (18.5)	23.0 (30.3)	**0.001**^**b**^
**AST**	< 46 U/l	22.0 (14.0)	29.0 (19.5)	**0.01**^**b**^
**ALT**	< 50 U/l	25.0 (8.0)	26.0 (17.5)	0.25^b^
**PT**	70–125%	101.9 ± 11.0	98.6 ± 16.2	0.32^a^
**Ferritin**	30–300 μg/l	133.0 (150.5)	261.0 (731.3)	**0.02**^**b**^
**Transferrin**	2.0–3.6 g/l	2.1 ± 0.3	1.8 ± 0.3	**< 0.0001**^**a**^
**TS**	16–45%	26.0 ± 9.6	70.1 ± 24.9	**< 0.0001**^**a**^
**Iron**	14–32 μmol/l	13.5 ± 4.7	31.8 ± 10.2	**< 0.0001**^**a**^
**Hepatic steatosis**	none	18 (90%)	16 (29.6%)	**< 0.0001**^**c**^
	mild	0 (0%)	18 (33.3%)	
	moderate	2 (10%)	12 (22.2%)	
	severe	0 (0%)	5 (9.3%)	
**FibroScan**	< 6 kPa	–	6.7 ± 4.8	–
**HFE-genotype**	C282Y	–	54 (100%)	–
***PNPLA3*genotype**	CC	–	26 (48.2%)	–
	CG	–	24 (44.4%)	–
	GG	–	4 (7.4%)	–

*ALT* alanine aminotransferase; *AST* aspartate aminotransferase; *BMI* body mass index; *HDL* high-density lipoprotein; *HH* hereditary hemochromatosis; *IQR* interquartile range; *LDL* low-density lipoprotein; *PNPLA3* human patatin like phospholipase domain-containing 3; *PT* prothrombin time; *SD* Standard deviation; *TS* transferrin saturation;

### Serum phospho- and sphingolipids of study population

In addition to serum neutral lipids and lipoproteins, we also determined serum contents of polar lipids (phospho- and sphingolipids) in 53 subjects with HH which were compared with those of healthy control group (Table 2). A significant difference was observed for total (*p* = 0.005) and polyunsaturated fatty acids (PUFA, *p* = 0.008) containing PE and total (*p* = 0.04) and monounsaturated fatty acids (MUFA, *p* = 0.01) containing PI with higher values in subjects with HH. No difference was observed for total, MUFA and PUFA containing PS between the two groups. In HH group, significant higher levels were observed PUFA containing LPE (*p* = 0.045). In LPC, a significant difference was seen in MUFA (*p* = 0.02) and PUFA (*p* = 0.004) containing LPC. No significance was reached for total and saturated LPC. Saturated (*p* < 0.0001), PUFA (*p* = 0.003), alkyl containing (*p* = 0.045) and total PC (*p* = 0.04) showed significant differences between the groups. Saturated (*p* = 0.03), total (*p* = 0.03) and MUFA (*p* = 0.045) containing SM were significantly elevated in HH group. PC/PE ratio was clearly lower in HH group indicating a shift towards PE and therefore indicating a disturbance in PC metabolism.

**Table 2:** Phospho- and sphingolipids (ng/μl) of study population. Serum samples were quantified in ng/μl serum. Mean ± SD or median (IQR) are shown with *p*-values for the group differences based on Welch’s t-test (a) or Mann-Whitney U test (b)

		Healthy *n* = 20	HH *n* = 53	***p***-value
***Phospholipids***				
**PC**	Saturated	996.2 ± 369.3	750.6 ± 30.6	**< 0.0008**^**a**^
	MUFA	2327.0 ± 866.7	2401.8 ± 976.2	0.75^a^
	PUFA	6163.9 ± 2104.8	8001.3 ± 2334.6	**0.003**^**a**^
	Alkyl	521.6 ± 169.7	619.3 ± 203.1	**0.045**^**a**^
	**Total**	10,008.8 ± 3154.7	11,773.0 ± 3339.3	**0.04**^**a**^
**LPC**	Saturated	51.5 ± 11.3	56.9 ± 14.6	0.10^a^
	MUFA	12.5 ± 3.4	10.5 ± 2.7	**0.02**^**a**^
	PUFA	19.6 ± 4.2	16.2 ± 4.2	**0.004**^**a**^
	**Total**	83.7 ± 17.6	83.6 ± 19.7	0.98^a^
**PI**	MUFA	14.4 ± 6.5	19.7 ± 10.0	**0.01**^**a**^
	PUFA	137.4 ± 50.1	165.0 ± 61.0	0.06^a^
	**Total**	152.1 ± 55.7	184.7 ± 69.1	**0.04**^**a**^
**PS**	MUFA	0.4 (2.6)	0.0 (4.0)	0.71^b^
	PUFA	17.7 (17.9)	24.6 (52.0)	0.15^b^
	**Total**	18.4 (18.3)	26.5 (59.5)	0.18^b^
**PE**	MUFA	75.2 (55.4)	91.9 (329.7)	0.05^b^
	PUFA	198.6 (103.5)	289.2 (257.0)	**0.005**^**b**^
	**Total**	276.4 (123.2)	407.8 (573.2)	**0.008**^**b**^
**LPE**	Saturated	1.2 (0.9)	1.3 (0.8)	0.87^b^
	MUFA	0.5 (0.4)	0.5 (0.9)	0.95^b^
	PUFA	1.6 (0.8)	2.3 (3.0)	**0.045**^**b**^
	**Total**	3.6 (1.7)	4.5 (4.8)	0.10^b^
***Sphingolipids***				
**SM**	Saturated	711.3 ± 271.3	876.1 ± 279.5	**0.03**^**a**^
	MUFA	2168.3 ± 1339.6	2879.7 ± 1173.2	**0.045**^**a**^
	PUFA	1537.9 ± 517.6	1728.4 ± 380.7	0.15^a^
	Alkyl	344.3 ± 256.5	465.4 ± 236.7	0.08^a^
	**Total**	4761.8 ± 2104.7	5949.6 ± 1743.3	**0.03**^**a**^
**Ratio PC/PE**		34.7	22.2	

*HH* hereditary hemochromatosis; *IQR* interquartile range; *LPC* lysophosphatidylcholine; *LPE* lysophosphatidylethanoletamine; *MUFA* monounsaturated fatty acid; *PC* phosphatidylcholine; *PE* phosphatidylethanolamine; *PS* phosphatidylserine; *PUFA* polyunsaturated fatty acid; *SD* standard deviation; *SM* sphingomyelin;

### *PNPLA3* genotype of study population

54 subjects with HH were analyzed according to their I148M *PNPLA3* polymorphism (rs738409). 48.2% were wildtype (CC), 44.4% were heterozygous (CG) and 7.4% homozygous (GG) for the *PNPLA3* polymorphism I148M (Table 1). Analysis of CC in comparison to CG/GG showed also no significant difference between the groups, especially in lipid (cholesterol, HDL, LDL, triglycerides) and iron parameters (transferrin, iron, TS, ferritin), grade of hepatic steatosis and fibrosis (Table 3). Phospho- and sphingolipids as well demonstrated no significant difference between these two groups (Table 4).

**Table 3:** *PNPLA3* genotype CC vs. CG/GG of hereditary hemochromatosis (HH) group. Mean ± SD or median (IQR) or frequencies n (%) are shown with p-values for the group differences based on Welch’s t-test (a), Mann-Whitney U test (b) or chi-square test (c)

	limits of normal	CC *n* = 26	CG/GG *n* = 28	***p***-value
**Cholesterol**	mg/dl	181.6 ± 35.2	193.9 ± 40.8	0.26^a^
**HDL**	> 40 mg/dl	58.8 ± 20.4	51.1 ± 18.1	0.18^a^
**LDL**	< 160 mg/dl	105.8 ± 22.7	116.3 ± 29.3	0.20^a^
**Triglycerides**	< 150 mg/dl	97.0 (58.8)	128.5 (125.0)	0.17^b^
**Ferritin**	μg/l	153.5 (465.5)	562.0 (1064.5)	0.18^b^
**Transferrin**	2.0–3.6 g/l	1.9 ± 0.3	1.8 ± 0.3	0.40^a^
**TS**	16–45%	73.8 ± 22.8	68.4 ± 26.7	0.44^a^
**Iron**	14–32 μmol/l	33.1 ± 9.2	30.6 ± 11.1	0.34^a^
**Albumin**	30–50 g/l	44.7 ± 2.7	44.9 ± 2.6	0.79^a^
**Bilirubin**	< 1.0 mg/dl	0.6 (0.5)	0.8 (0.7)	0.20^b^
**GGT**	< 60 U/l	23.0 (39.5)	26.0 (25.3)	0.94^b^
**AST**	< 46 U/l	25.0 (15.0)	35.5 (20.3)	**0.04**^**b**^
**ALT**	< 50 U/l	23.5 (10.5)	31.5 (16.5)	0.19^b^
**BMI**	kg/m^2^	26.4 ± 3.9	25.7 ± 2.7	0.44^a^
**FibroScan**	< 6 kPa	6.8 ± 5.4	6.5 ± 4.1	0.79^a^
**Hepatic steatosis**	none	9 (34.6%)	7 (25%)	0.62^c^
	mild	9 (34.6%)	9 (32.1%)	
	moderate	4 (15.4%)	8 (28.6%)	
	severe	3 (3.8%)	2 (7.1%)	

*ALT* alanine aminotransferase; *AST* aspartate aminotransferase; *BMI* body mass index; *HDL* high-density lipoprotein; *IQR* interquartile range; *LDL* low-density lipoprotein; *PNPLA3*. human patatin like phospholipase domain-containing 3; *SD* standard deviation; *TS* transferrin saturation;

**Table 4:** Phospho- and sphingolipids (ng/μl) of hereditary hemochromatosis (HH) group stratified by *PNPLA3* genotype. Serum samples were quantified in ng/μl serum. Mean ± SD or median (IQR) are shown with p-values for the group differences based on Welch’s t-test (a) or Mann-Whitney U test (b)

		CC *n* = 26	CG/GG *n* = 28	***p***-value
***Phospholipids***				
**PC**	Saturated	753.7 ± 37.6	747.8 ± 23.0	0.50^a^
	MUFA	2524.5 ± 1078.8	2292.3 ± 880.0	0.40^a^
	PUFA	8352.1 2 ± 467.5	7688.1 ± 2206.7	0.31^a^
	Alkyl	619.1 ± 204.9	619.4 ± 205.3	0.99^a^
	**Total**	12,249.3 ± 3624.0	11,347.7 ± 3066.5	0.34^a^
**LPC**	Saturated	54.0 ± 12.4	59.5 ± 16.0	0.17^a^
	MUFA	10.2 ± 2.5	10.8 ± 2.9	0.44^a^
	PUFA	15.8 ± 4.0	16.6 ± 4.5	0.52^a^
	**Total**	80.0 ± 16.5	86.8 ± 21.9	0.21^a^
**PI**	MUFA	18.80 ± 9.5	20.5 ± 10.5	0.55^a^
	PUFA	155.1 ± 57.6	173.9 ± 63.7	0.26^a^
	**Total**	173.9 ± 64.9	194.3 ± 72.4	0.28^a^
**PS**	MUFA	3.2 (5.1)	0.0 (2.6)	0.16^b^
	PUFA	24.6 (98.3)	23.4 (39.4)	0.55^b^
	**Total**	27.2 (104.1)	23.6 (49.9)	0.43^b^
**PE**	MUFA	85.7 (363.4)	92.8 (271.3)	0.99^b^
	PUFA	279.1 (236.7)	302.5 (278.7)	0.48^b^
	**total**	370.9 (607.0)	409.1 (545.4)	0.64^b^
**LPE**	Saturated	1.3 (0.8)	1.3 (0.8)	0.94^b^
	MUFA	0.4 (1.1)	0.6 (0.9)	0.46^b^
	PUFA	2.4 (4.7)	2.2 (2.5)	0.76^b^
	**Total**	3.9 (5.1)	4.6 (4.5)	0.78^b^
***Sphingolipids***				
**SM**	Saturated	892.4 ± 269.5	861.6 ± 292.2	0.69^a^
	MUFA	3074.6 ± 1122.1	2705.7 ± 1210.4	0.26^a^
	PUFA	1702.8 ± 409.3	1751.3 ± 359.3	0.65^a^
	Alkyl	461.2 ± 228.5	469.10 ± 248.0	0.90^a^
	**Total**	6130.94 ± 1688.2	5787.7 ± 1806.0	0.48^a^
**Ratio PC/PE**		23.1	21.4	

*HH* hereditary hemochromatosis; *IQR* interquartile range; *LPC* lysophosphatidylcholine; *LPE* lysophosphatidylethanoletamine; *PC* phosphatidylcholine; *PE* phosphatidylethanolamine; *PS* phosphatidylserine; *SD* standard deviation; *SM* sphingomyelin;

## Discussion

In our study, we analyzed lipid metabolism and hepatic steatosis in HH. This study is to our knowledge the first study in HH which investigated in addition to lipoproteins (HDL, LDL) and neutral lipids (cholesterol, triglycerides) phospho- and sphingolipid metabolism. Our results strengthen the hypothesis of altered lipid metabolism in HH, especially in phospholipid metabolism and susceptibility to NAFLD and this was independent of *PNPLA3* polymorphism.

It is known that hepatic steatosis is a cofactor in liver injury in HH [22], and HH-dependent susceptibility to NAFLD is independent of BMI and triglyceride levels [23, 24]. Clinical significant liver disease in HH is also associated with risk factors like heavy alcohol abuse and concomitant chronic liver diseases like chronic hepatitis C [37]. As HH subjects with high (> 20 g/day) alcohol consumption and concomitant chronic liver diseases were excluded from our study, it has to be assumed that these risk factors do not play a role in the observed alterations in our cohort.

In our cohort, BMI was as well not affected in HH, and this indicates non-obese NAFLD in HH pathogenesis. However, our cohort shows that blood triglycerides were significantly higher and hepatic steatosis was significantly more frequent in HH group. While the role of HFE polymorphism in the occurrence of NAFLD still remains unclear, our findings may suggest that iron-overload and subsequent oxidative stress in HH could be due to the alteration of hepatic triglyceride metabolism associated with hepatic steatosis. Accordingly, it is known that iron-overload increases serum triglycerides associated with fatty liver in experimental animals [38]. NAFLD patients also show an increase of both triglycerides and iron in the liver [39], and that iron stores as ferritin in NAFLD is associated with hypertriglyceridemia [40]. Iron overload can also affect visceral adipose tissue metabolism by a mechanism involving hepcidin up-regulation [41]. In addition to NAFLD, hepatocellular iron deposition is associated with an increased risk of hepatic fibrosis [42] suggesting a critical role of iron in NAFLD progression to NASH. Taken together, increased blood triglycerides observed in our HH cohort is a pathologic marker of exaggerated hepatic steatosis caused by iron-overload. The alteration of adipose tissue metabolism by iron-overload could result in the lack of any increase of BMI in HH, despite of increased hepatic steatosis.

Together with increased hepatic triglycerides, hepatic steatosis is associated with the depletion of liver phospholipids including PC and PE in morbidly obese mice [43] and NAFLD patients [44]. Studies in *PEMT*-deficient mice have indicated that a decrease in hepatic PC/PE ratio is linked to NAFLD [45], and variants of *PEMT* gene is associated with non-obese NAFLD [46]. HH subjects in our study had significantly higher serum PUFA-containing and total PE species and lower saturated-containing PC resulting in a decrease of PC/PE ratio. Thus, hepatic metabolism in steatotic HH may be dominated by a shift of metabolism towards PE as seen in *PEMT*-deficient mice [45] and non-obese NAFLD [46]. In support of our results, patients with NAFLD and NASH show higher hepatic PE and lower erythrocyte PC [47]. Furthermore, serum PE is shown to be significantly increased in NASH but not in NAFLD [48] Thus, a decrease of serum PC/PE ratio appears to be a hallmark biomarker of HH with iron-overload being critical in the progression to NASH [49]. While iron deficiency is reported to increase hepatic PC and PE in rats [50], conversely, iron-overload in HH liver may induce a decrease of PC and PE differently by an unknown mechanism. As liver X receptor (LXR) regulates PUFA metabolism in phospholipids [51] particularly PE [52], we speculate that LXR could be a target of iron-induced oxidative stress [53] that could lead to increased hepatic and serum PE in steatotic HH.

While carriers of *PNPLA3* polymorphism (rs738409/I148M) are associated with hepatic steatosis and impaired lipid metabolism [25, 28–30, 32] and hepatic steatosis was frequently seen in our HH cohort, we analyzed *PNPLA3* polymorphism. In our cohort, *PNPLA3* polymorphism did not show any significant alterations on iron and lipid metabolism parameters including lipoproteins (HDL, LDL), neutral lipids (cholesterol, triglycerides), phospho- and sphingolipid metabolism. In a recent study, the I148M variant in HH was also not associated with altered lipid levels, but with the presence of hepatic steatosis and severity of fibrosis and therefore maybe representing a potential factor for fibrosis progression [34]. In our cohort, no effect on grade of hepatic steatosis and fibrosis for risk allele G was seen indicating that it does not have any impact on NAFLD pathophysiology in HH. Hepatic steatosis in HH is therefore rather related to iron-overload and subsequent oxidative stress with impaired apolipoprotein B100 (ApoB100) and VLDL secretion [9, 10]. Our study is to our knowledge the first study which investigated phospholipid metabolism in HH depending on *PNPLA3* polymorphism. Our HH cohort revealed significantly lower serum PC and significantly higher serum PE when compared to healthy controls, but this was independent of *PNPLA3* polymorphism suggesting that it does not have any effect on altered phospholipid metabolism in HH.

Limitations of our study are a small cohort size. For further investigations, it is important to analyze liver lipidome, measure VLDL secretion and phospholipid profiles of VLDL of HH patients to clarify the role of altered VLDL secretion and composition in pathogenesis of susceptibility to NAFLD in HH.

## Conclusion

Our study supports the hypothesis of altered lipid metabolism and susceptibility to NAFLD in HH. Disturbed phospholipid metabolism is likely an important factor in pathogenesis of hepatic steatosis in HH.

## Data Availability

The dataset used and analyzed during the current study is available from the corresponding author on reasonable request.

## Abbreviations

ALT: Alanine aminotransferase
ApoB100: Apolipoprotein B100
AST: Aspartate aminotransferase
BMI: Body mass index
FFA: Free fatty acids
FP-1: Ferroportin-1
GGT: Gamma-glutamyltransferase
HH: Hereditary hemochromatosis
HDL: High-density lipoprotein
LCPUFA: Long-chain polyunsaturated fatty acids
LDL: Low-density lipoprotein
LPC: Lysophosphatidylcholine
LPE: Lysophosphatidylethanolamine
LXR: Liver X receptor
MUFA: Monounsaturated fatty acid
NAFLD: Non-alcoholic fatty liver disease
NASH: Non-alcoholic steatohepatitis
PC: Phosphatidylcholine
PE: Phosphatidylethanolamine
PEMT: Phosphatidylethanolamine N-methyltransferase
PI: Phosphatidylinositol
PNPLA3: Human patatin like phospholipase domain-containing 3
PS: Phosphatidylserine
PT: Prothrombin time
PUFA: Polyunsaturated fatty acid
SM: Sphingomyelin
TS: Transferrin saturation
VLDL: Very-low density lipoprotein

## References

[1] Allen KJ, Gurrin LC, Constantine CC, Osborne NJ, Delatycki MB, Nicoll AJ (2008). Iron-overload–related disease in HFE hereditary hemochromatosis. N Engl J Med.

[2] Adams PC, Reboussin DM, Barton JC, McLaren CE, Eckfeldt JH, McLaren GD (2005). Hemochromatosis and Iron overload screening (HEIRS) study research investigators. Hemochromatosis and iron-overload screening in a racially diverse population. N Engl J Med.

[3] Bloomer SA, Brown KE (2019). Iron-induced liver injury: a critical reappraisal. Int J Mol Sci.

[4] Fleming RE, Ponka PN (2012). Iron overload in human disease. Engl J Med.

[5] Ahmed U, Latham PS, Oates PS (2012). Interactions between hepatic iron and lipid metabolism with possible relevance to steatohepatitis. World J Gastroenterol.

[6] Brunet S, Thibault L, Delvin E, Yotov W, Bendayan M, Levy E (1999). Dietary iron overload and induced lipid peroxidation are associated with impaired plasma lipid transport and hepatic sterol metabolism in rats. Hepatology..

[7] Graham RM, Chua AC, Carter KW, Delima RD, Johnstone D, Herbison CE (2010). Hepatic iron loading in mice increases cholesterol biosynthesis. Hepatology..

[8] Choi JS, Koh IU, Lee HJ, Kim WH, Song J (2013). Effects of excess dietary iron and fat on glucose and lipid metabolism. J Nutr Biochem.

[9] Barisani D, Meneveri R, Ginelli E, Cassani C, Conte D (2000). Iron overload and gene expression in HepG2 cells: analysis by differential display. FEBS Lett.

[10] Pankow JS, Boerwinkle E, Adams PC, Guallar E, Leiendecker-Foster C, Rogowski J (2008). HFE C282Y homozygotes have reduced low-density lipoprotein cholesterol: the Atherosclerosis Risk in Communities (ARIC) Study. Transl Res.

[11] Cabrita M, Pereira CF, Rodrigues P, Cardoso EM, Arosa FA. Altered expression of CD1d molecules and lipid accumulation in the human hepatoma cell line HepG2 after iron loading FEBS J. 2005;272(1):152–165. https://doi.org/10.1111/j.1432-1033.2004.04387.x. 10.1111/j.1432-1033.2004.04387.x 15634340

[12] Du Plooy JN, Bester J, Pretorius E (2018). Eryptosis in Haemochromatosis: implications for rheology. Clin Hemorheol Microcirc.

[13] Stechemesser L, Eder SK, Wagner A, Patsch W, Feldman A, Strasser M (2016). Metabolomic profiling identifies potential pathways involved in the interaction of iron homeostasis with glucose metabolism. Mol Metab.

[14] Noga AA, Vance DE (2003). A gender-specific role for phosphatidylethanolamine N-methyltransferase-derived phosphatidylcholine in the regulation of plasma high density and very low density lipoproteins in mice. J Biol Chem.

[15] Jacobs RL, Devlin C, Tabas I, Vance DE (2004). Targeted deletion of hepatic CTP:phosphocholine cytidylyltransferase alpha in mice decreases plasma high density and very low density lipoproteins. J Biol Chem.

[16] Valenzuela R, Rincón-Cervera MÁ, Echeverría F, Barrera C, Espinosa A, Hernández-Rodas MC (2018). Iron-induced pro-oxidant and pro-lipogenic responses in relation to impaired synthesis and accretion of long-chain polyunsaturated fatty acids in rat hepatic and extrahepatic tissues. Nutrition..

[17] Mateo-Gallego R, Calmarza P, Jarauta E, Burillo E, Cenarro A, Civeira F (2010). Serum ferritin is a major determinant of lipid phenotype in familial combined hyperlipidemia and familial hypertriglyceridemia. Metabolism..

[18] Solanas-Barca M, Mateo-Gallego R, Calmarza P, Jarauta E, Bea AM, Cenarro A (2009). Mutations in HFE causing hemochromatosis are associated with primary hypertriglyceridemia. J Clin Endocrinol Metab.

[19] Casanova-Esteban P, Guiral N, Andrés E, Gonzalvo C, Mateo-Gallego R, Giraldo P (2011). Effect of phlebotomy on lipid metabolism in subjects with hereditary hemochromatosis. Metabolism..

[20] Mateo-Gallego R, Lacalle L, Pérez-Calahorra S, Marco-Benedí V, Recasens V, Padrón N (2018). Efficacy of repeated phlebotomies in hypertriglyceridemia and iron overload: a prospective, randomized, controlled trial. J Clin Lipidol.

[21] Cash WJ, O'Neill S, O'Donnell ME, McCance DR, Young IS, McEneny J (2014). Endothelial function, antioxidant status and vascular compliance in newly diagnosed HFE C282Y homozygotes. Adv Med Sci.

[22] Powell EE, Ali A, Clouston AD, Dixon JL, Lincoln DJ, Purdie DM (2005). Steatosis is a cofactor in liver injury in hemochromatosis. Gastroenterology..

[23] Valenti L, Dongiovanni P, Fracanzani AL, Santorelli G, Fatta E, Bertelli C, Taioli E, Fiorelli G, Fargion S (2003). Increased susceptibility to nonalcoholic fatty liver disease in heterozygotes for the mutation responsible for hereditary hemochromatosis. Dig Liver Dis.

[24] Ye Q, Qian BX, Yin WL, Wang FM, Han T (2016). Association between the HFE C282Y, H63D polymorphisms and the risks of non-alcoholic fatty liver disease, liver cirrhosis and hepatocellular carcinoma: an updated systematic review and meta-analysis of 5,758 cases and 14,741 controls. PLoS One.

[25] Kienesberger PC, Oberer M, Lass A, Zechner R (2009). Mammalian patatin domain containing proteins: a family with diverse lipolytic activities involved in multiple biological functions. J Lipid Res.

[26] Romeo S, Kozlitina J, Xing C, Pertsemlidis A, Cox D, Pennacchio LA, et al. Genetic variation in PNPLA3 confers susceptibility to nonalcoholic fatty liver disease. Nat Genet 2008;40:1461–1465. https://doi.org/10.1038/ng.257. 10.1038/ng.257 PMC2597056 18820647

[27] Eslam M, Valenti L, Romeo S (2018). Genetics and epigenetics of NAFLD and NASH: clinical impact. J Hepatol.

[28] Pingitore P, Pirazzi C, Mancina RM, Motta BM, Indiveri C, Pujia A, Montalcini T, Hedfalk K, Romeo S (2014). Recombinant PNPLA3 protein shows triglyceride hydrolase activity and its I148M mutation results in loss of function. Biochim Biophys Acta.

[29] Sookoian S, Pirola CJ (2011). Meta-analysis of the influence of I148M variant of patatin-like phospholipase domain containing 3 gene (PNPLA3) on the susceptibility and histological severity of nonalcoholic fatty liver disease. Hepatology..

[30] He S, McPhaul C, Li JZ, Garuti R, Kinch L, Grishin NV (2010). A sequence variation (I148M) in PNPLA3 associated with nonalcoholic fatty liver disease disrupts triglyceride hydrolysis. J Biol Chem.

[31] Pirazzi C, Adiels M, Burza MA, Mancina RM, Levin M, Ståhlman M (2012). Patatin-like phospholipase domaincontaining 3 (PNPLA3) I148M (rs7,38,409) affects hepatic VLDL secretion in humans and in vitro. J Hepatol.

[32] Ruhanen H, Perttilä J, Hölttä-Vuori M, Zhou Y, Yki-Järvinen H, Ikonen E, Käkelä R, Olkkonen VM (2014). PNPLA3 mediates hepatocyte triacylglycerol remodeling. J Lipid Res.

[33] Karamfilova V, Gateva A, Assyov Y, Alexiev A, Savov A, Yaneva N, Ivanova I, Ivanova-Boyanova R, Ivanova R, Vlahova Z, Mateva L, Kamenov Z (2019). PNPLA3 I148M polymorphism in patients with nonalcoholic fatty liver disease, obesity and Prediabetes. J Gastrointestin Liver Dis.

[34] Speliotes EK, Butler JL, Palmer CD, Voight BF, Hirschhorn JN (2010). PNPLA3 variants specifically confer increased risk for histologic nonalcoholic fatty liver disease but not metabolic disease. Hepatology..

[35] Valenti L, Maggioni P, Piperno A, Rametta R, Pelucchi S, Mariani R, Dongiovanni P, Fracanzani AL, Fargion S (2012). Patatin-like phospholipase domain containing-3 gene I148M polymorphism, steatosis, and liver damage in hereditary hemochromatosis. World J Gastroenterol.

[36] Jiao L, Gan-Schreier H, Zhu X, Wei W, Tuma-Kellner S, Liebisch G (2017). Ageing sensitized by iPLA2β deficiency induces liver fibrosis and intestinal atrophy involving suppression of homeostatic genes and alteration of intestinal lipids and bile acids. Biochim Biophys Acta.

[37] Sanchez-Luna SA, Brown KE (2017). Clinical Burden of Liver Disease From Hemochromatosis at an Academic Medical Center. Hepatol Commun.

[38] Silva M, Silva ME, de Paula H, Carneiro CM, Pedrosa ML (2008). Iron overload alters glucose homeostasis, causes liver steatosis, and increases serum triacylglycerols in rats. Nutr Res.

[39] Martin-Rodriguez JL, Gonzalez-Cantero J, Gonzalez-Cantero A, Martí-Bonmatí L, Alberich-Bayarri Á, Gonzalez-Cejudo T, Gonzalez-Calvin JL (2019). Insulin resistance and NAFLD: relationship with intrahepatic iron and serum TNF-α using 1H MR spectroscopy and MRI. Diabetes Metab.

[40] Suárez-Ortegón MF, Ensaldo-Carrasco E, Shi T, McLachlan S, Fernández-Real JM, Wild SH (2018). Ferritin, metabolic syndrome and its components: a systematic review and meta-analysis. Atherosclerosis..

[41] Dongiovanni P, Ruscica M, Rametta R, Recalcati S, Steffani L, Gatti S, Girelli D, Cairo G, Magni P, Fargion S, Valenti L (2013). Dietary iron overload induces visceral adipose tissue insulin resistance. Am J Pathol.

[42] O'Brien J, Powell LW (2012). Non-alcoholic fatty liver disease: is iron relevant?. Hepatol Int.

[43] Deng X, Wang J, Jiao L, Utaipan T, Tuma-Kellner S, Schmitz G, Liebisch G, Stremmel W, Chamulitrat W (2016). iPLA2β deficiency attenuates obesity and hepatic steatosis in Ob/Ob mice through hepatic fatty-acyl phospholipid remodeling. Biochim Biophys Acta.

[44] Puri P, Baillie RA, Wiest MM, Mirshahi F, Choudhury J, Cheung O (2007). A lipidomic analysis of nonalcoholic fatty liver disease. Hepatology..

[45] Li Z, Agellon LB, Allen TM, Umeda M, Jewell L, Mason A, Vance DE (2006). The ratio of phosphatidylcholine to phosphatidylethanolamine influences membrane integrity and steatohepatitis. Cell Metab.

[46] Bale G, Vishnubhotla RV, Mitnala S, Sharma M, Padaki RN, Pawar SC, Duvvur RN (2019). Whole-exome sequencing identifies a variant in Phosphatidylethanolamine N-methyltransferase gene to be associated with lean-nonalcoholic fatty liver disease. J Clin Exp Hepatol.

[47] Arendt BM, Ma DW, Simons B, Noureldin SA, Therapondos G, Guindi M, Sherman M, Allard JP (2013). Nonalcoholic fatty liver disease is associated with lower hepatic and erythrocyte ratios of phosphatidylcholine to phosphatidylethanolamine. Appl Physiol Nutr Metab.

[48] Ma DW, Arendt BM, Hillyer LM, Fung SK, McGilvray I, Guindi M, Allard JP (2016). Plasma phospholipids and fatty acid composition differ between liver biopsy-proven nonalcoholic fatty liver disease and healthy subjects. Nutr Diabetes.

[49] Tsurusaki S, Tsuchiya Y, Koumura T, Nakasone M, Sakamoto T, Matsuoka M, Imai H, Yuet-Yin Kok C, Okochi H, Nakano H, Miyajima A, Tanaka M (2019). Hepatic ferroptosis plays an important role as the trigger for initiating inflammation in nonalcoholic steatohepatitis. Cell Death Dis.

[50] Stangl GI, Kirchgessner M (1998). Different degrees of moderate iron deficiency modulate lipid metabolism of rats. Lipids..

[51] Jalil A, Bourgeois T, Ménégaut L, Lagrost L, Thomas C, Masson D (2019). Revisiting the Role of LXRs in PUFA Metabolism and Phospholipid Homeostasis. Int J Mol Sci.

[52] Santinha D, Klopot A, Marques I, Ellis E, Jorns C, Johansson H, Melo T, Antonson P, Jakobsson T, Félix V, Gustafsson JÅ, Domingues MR, Mode A, Helguero LA (2019). Lipidomic analysis of human primary hepatocytes following LXR activation with GW3965 identifies AGXT2L1 as a main target associated to changes in phosphatidylethanolamine. J Steroid Biochem Mol Biol.

[53] Alba G, Reyes ME, Santa-María C, Ramírez R, Geniz I, Jiménez J, Martín-Nieto J, Pintado E, Sobrino F (2012). Transcription of liver X receptor is down-regulated by 15-deoxy-Δ(12,14)-prostaglandin J(2) through oxidative stress in human neutrophils. PLoS One.

